# Behavioral duality in an integrated agent

**DOI:** 10.3389/fnhum.2014.00614

**Published:** 2014-08-08

**Authors:** Ivan Martinez-Valbuena, Javier Bernacer

**Affiliations:** Mind-Brain Group, Institute for Culture and Society, University of NavarraPamplona, Spain

**Keywords:** Kahneman, habits, goal-directed actions, consciousness, cognitive control, prefrontal cortex

Humans can consolidate and carry out habits other animals cannot. This statement is mainly sustained by the fact that humans have a unique cognitive control of their actions: we can let our attention fade away to perform automatic tasks more efficiently, we can detect if there has been an unexpected problem in their implementation, and we can regain conscious control of the action if necessary. We tend to dichotomize this cognitive process into two “systems,” namely goal-directed versus habitual (Dickinson, 1985), conscious versus unconscious (Crick and Koch, 1998), or slow versus fast (Kahneman, 2011). If we just put it in those terms, these two ways of tackling the challenges of a changing environment seem to be present in non-human animals. However, all dichotomies imply a difficulty to deal with: the regulation of the transition between the two systems. Is this carried out by a third element, or regulated by one of the systems? Could it be more convenient to view it as a continuum, rather than a dichotomy? In any case, we believe this transition has a level of complexity in humans that makes it qualitatively different from its analog in animals. In fact, this “cognitive bridge” might be a major feature to characterize a reliable behavior, since a particular task or problem is more efficiently tackled when the transition between the two systems is more adequate. Moreover, the integrity of this link could be an indicator to detect prodromal psychiatric conditions, as it has been suggested for slips-of-action (Gillan et al., 2011).

In order to justify these ideas, we will focus first on Kahneman's distinction between systems 1 and 2 (Kahneman, 2011). On the one hand, System 1 is responsible for making decisions rapidly. The purpose of this system is to give us an assessment of the environment around us as quickly as possible so that we are able to respond as fast as possible. To perform this task, System 1 follows general rules or guidelines (heuristics). In all, System 1 is intended to help us make decisions more quickly, and is very useful (let's say “just fine”) in most cases. However, one of the characteristics of these decisions is the lack of voluntary control, what is a problem considering this system is responsible of many of the decisions and judgments we make. Given to its “automatic” nature, System 1 also has biases and systematic errors that are likely to happen in some situations.

On the other hand, System 2 acts when a problem which System 1 has no solution for arises. System 2, apparently, can take control of the whole process at any time. It is somewhat triggered by some external or internal alarm that draws its attention and makes it take “conscious” control of the situation. One of the problems of this system is that it is lazy and can be easily exhausted. Therefore, it usually accepts the decisions of System 1 without monitoring them. One proof of System 2's negligence is what Kahneman calls WYSIATI (“What You See Is All There Is”), a general rule that “facilitates the achievement of coherence and of the cognitive ease that causes us to accept a statement as true.” System 1 easily gets that coherence, and System 2 usually allows it to jump to conclusions and act. In different sets of experiments, Kahneman demonstrates that humans are not good at all with statistics or handling mathematics; in his opinion, this is because humans simplify judgments to make them more understandable and deal with them just through heuristics that System 1 can handle. This general view of humans as poor rational decision-makers is also supported by other authors (see, for example, Ariely, 2008).

In our opinion, this division of human cognition into two systems fits well with the usual opposition between goal-directed versus habitual systems (Dickinson, 1985). In general, goal-directed actions are viewed as conscious, flexible, and sensitive to outcome devaluation, whereas habits are mainly unconscious, rigid and insensitive to changes in the value of the outcome. The features of goal-directed and habit systems were mainly drawn from studies in animals. The typical experiment about this subject consists on teaching the contingency between an instrumental action (for example, a lever press) and a reward to the animal (Adams and Dickinson, 1981). At the beginning, the animal's behavior is goal directed, and it performs the action to obtain the reward. However, this behavior becomes “habitual” (in this context, a motor routine) after many repetitions. When that happens, the value of the reward is transferred to the lever press itself: even though the outcome is devalued (gets the animal sick) or the animal is sated, it keeps pressing the lever. This is why habits have been opposed to goal-directed behavior.

A quick look suggests that habits and goal-directed actions are intimately related to Systems 1 and 2, respectively. This is also supported by the identification of the goal-directed system with a model-based reinforcement learning scheme, since it can be viewed “in terms of sophisticated, computationally demanding, prospective planning, in which a decision tree of possible future states and actions is built using a learned internal model of the environment” (Dolan and Dayan, 2013). The habitual system, on the other hand, follows a model-free scheme, which “is computationally efficient, since it replaces computation (i.e., the burdensome simulation of future states) with memory (i.e., stored discounted values of expected future reward); however, the forward-looking nature of the prediction error makes it statistically inefficient” (Dolan and Dayan, 2013). Following these analogies between systems, we can assume that some actions that at the beginning fall under the domain of System 2 might be transferred to System 1 through learning, like goal-directed actions become habits through experience.

Concerning the neural bases of these systems, the striatum and its cortical afferents and –indirect– target areas in the cortex play a major role. It is widely accepted that the cognitive part of the striatum –caudate nucleus and anterior putamen– are involved in the planning and execution of goal-directed actions, together with the prefrontal cortex (Balleine et al., 2007). On the other hand, the sensorimotor striatal aspects –mainly the posterior putamen– and the supplementary motor area of the cortex are particularly active when the agent is performing a well-learned action (Miyachi et al., 2002; Ashby et al., 2010). Furthermore, the activity of the neurons in these areas follows a “chunked” pattern: they are mainly active at certain stages of the motor routine (for example at the beginning and the end of the sequence, when a particular switch or turn is needed, etc), and this activity is reduced in the rest of the motor sequence (Graybiel, 1998). Although some authors question a sharp neuroanatomical basis of Kahneman's Systems 1 and 2 (Gold and Shadlen, 2007), our train of thought in this manuscript suggests that the more reflective System 2 should be based in the prefrontal cortex –both dorsal and ventral–, and the cognitive regions of the basal ganglia. Likewise, the more automatic System 1 would lie on motor and premotor cortical regions, as well as on the sensorimotor aspects of those subcortical nuclei.

This neuroscientific framework identifies the habit system with automaticity, rigidity and unconsciousness; however, we are intending to challenge this view in past and forthcoming contributions (Bernacer and Gimenez-Amaya, 2013; Bernacer et al., 2014). In a nutshell, we propose to view the phenomenon of action from the point of view of the agent as a whole, and not from an isolated movement. Hence, it could be more convenient to understand System 1 –or habits– as a resource of System 2, rather than as opposed systems in competition. Whereas a motor routine (i.e., what is commonly called “habit” in neuroscience) implies the sequential and unconscious performance of movements, they usually pursue the goal set by the agent. In fact, the more engrained the routine is, the easier for the agent to achieve that goal. Furthermore, the agent can consciously stop or correct the movement at any point, since the habit releases the higher cognitive regions of the brain to improve the performance of the action. A very simple example of this is a tennis service, which should be “goal directed” to place the ball wherever the player wants. It involves a set of movements such as throwing the ball upwards, moving the feet, putting the arm back, etc. Only when these motor routines are learned correctly, the player is able to concentrate on other aspects of the service such as the speed, spin, or exploiting the weaknesses of the receiver. This can be also exemplified with other kinds of habits such as driving, playing an instrument, tackling a mathematical problem, and so on. They all suggest that “automatic” routines are governed by higher cognitive functions to better achieve a particular goal.

Kahneman's Systems 1 and 2 allow as well this release of consciousness from everyday decisions to focus on more complicated situations. As Kahneman himself and other authors defend (Ariely, 2008), the problem arises when System 2 is rarely used or either system is applied to inadequate situations. However, we believe that the most effective agent does not exclusively rely on System 2, but efficiently uses all resources of “each system” in the right situation and, more importantly, carries out an appropriate transition between them. That is, in our opinion, a “rational” agent. This could be also said about goal-directed and habitual systems. Moreover, we believe that this transition between systems is subject to learning, and it can be performed more effectively through experience.

If we understand these fragmentations of cognition as independent systems in competition, we encounter an important problem: is there an additional mechanism in charge of the transition between systems, or is this regulated by one of the systems itself? If the first option were true, we would find the difficulty of defining the nature –both conceptually and anatomically– of a “third system” qualitatively different than the other two. This would lead us to an *ad infinitum* process –the need of a fourth element to regulate the activity of the third, and so on–, and therefore we believe this hypothesis should be rejected. Considering the second option, it seems that only the highly cognitive System 2 could be in charge of leading the transition between systems, which in turn dissolves a rigorous separation in two systems. The role of System 2 in leading the transition is clear when the agent decides to regain conscious control of a task generally performed in an unconscious manner. In this sense, the interaction of the orbitofrontal cortex with either the cognitive or sensorimotor aspects of the striatum plays a central role in shifting between goal-directed actions and motor routines (Gremel and Costa, 2013). In other situations, an external cue such as an error may set the alarm for System 2 to retake control of the action. Regarding this, the anterior cingulate cortex has been reported to be active in highly-conflictive decision making situations (Goñi et al., 2011); for that reason, some authors relate this cortical area with error monitoring (Carter, 1998; Botvinick et al., 2004). A recent report suggests a new model of reinforcement learning and conflict monitoring, which involves a wide network including different areas of the cortex (posterior parietal, precentral, anterior cingulate and prefrontal) and the basal ganglia (Zendehrouh et al., 2013).

To sum up, this opinion article suggests viewing Kahneman's systems as analogous to the goal-directed/habits dichotomy in order to improve the understanding of some aspects of human cognition. Further, we believe a strict separation between systems in competition is problematic, since System 2 is always in charge of governing the interplay between systems: therefore, System 1 –or habits– should be understood as a resource of System 2. This view could shed some light on the understanding of habits as a source of learning, plasticity and freedom for the agent. Finally, an inappropriate cognitive control of habits could be an indicator of certain psychiatric conditions.

## Conflict of interest statement

The authors declare that the research was conducted in the absence of any commercial or financial relationships that could be construed as a potential conflict of interest.
